# Pollinator-mediated selection on floral traits varies in space and between morphs in *Primula secundiflora*

**DOI:** 10.1093/aobpla/ply059

**Published:** 2018-10-01

**Authors:** Yun Wu, Tao Zhong, Zhi-Qiang Zhang, Qing-Jun Li

**Affiliations:** 1 School of Civil Engineering, Architecture and Environment, Xihua University, Chengdu, China; 2 CAS Key Laboratory of Tropical Forest Ecology, Xishuangbanna Tropical Botanical Garden, Chinese Academy of Sciences, Mengla, China; 3 University of Chinese Academy of Sciences, Beijing, China; 4 College of Landscape Architecture, Sichuan Agricultural University, Chengdu, China; 5 State Key Laboratory for Conservation and Utilization of Bio-Resources in Yunnan, Laboratory of Ecology and Evolutionary Biology, Yunnan University, Kunming, China

**Keywords:** Floral divergence, inter-morph variation, pollinator-mediated selection, *Primula secundiflora*, spatial variation

## Abstract

Elucidating how variation in selection shapes the evolution of flowers is key to understanding adaptive differentiation processes. We estimated pollinator-mediated selection through female function in L-morph (long-style and short-anther phenotype) and S-morph (short-style and long-anther phenotype) flowers among four *Primula secundiflora* populations with different pollinator assemblages. Variation in pollinator assemblage strongly contributed to differences in reproductive success among populations and between morphs of the primrose species. A wider corolla tube width was selected in the bumblebee-dominated populations, whereas shorter corolla tube length and wider corolla tube width were selected in the syrphid fly-dominated populations. Morph-specific variation in pollinator-mediated selection on corolla tube length was detected in the syrphid fly-dominated populations. A shorter corolla tube was selected in the L-morph flowers. However, similar selective pressure on this trait was not observed in the S-morph flowers. These results show that variation in pollinator assemblage leads to variation in selection in space and between morphs. The findings highlight the potential forces of different pollinator agents in driving floral evolution in this primrose species.

## Teaser

This paper researches the spatial and inter-morph variation in pollinator-mediated selection on floral traits in the heterostylous taxa, *Primula secundiflora*. Our results indicate that a wider corolla tube width was selected in the bumblebee-dominated populations, whereas shorter corolla tube length and wider corolla tube width were selected in the syrphid fly-dominated populations. Morph-specific variation in pollinator-mediated selection on corolla tube length was detected in the syrphid fly-dominated populations. The findings highlight the potential forces of different pollinator agents in driving floral evolution in this primrose species.

## Introduction

In angiosperms, diverse floral architectures are shaped by pollinators, and different pollinator–plant interactions may lead to variable pollinator-mediated selection on floral traits in space and time (Harder and Johnson 2009; Parachnowitsch and Kessler 2010; Sletvold and Ågren 2010; Siepielski *et al.* 2013; Van der Niet *et al.* 2014; Vlašánková *et al.* 2017). Heterostyly, a specific floral syndrome, that includes distyly and tristyly, has been shown to accelerate floral diversification in Primulaceae, Amaryllidaceae and Rubiaceae (Barrett and Shore 2008; Hodgins and Barrett 2008; Pérez-Barrales and Arroyo 2010; de Vos *et al.* 2014). Heterostyly may create complex and specific interactions between functional pollinator and flower that lead to differential pollinator-mediated selective pressures on floral traits. Studies that experimentally quantify the importance of pollinators as selective agents and how importance spatial and inter-morph varies are helpful to fully understand the role of pollinator-mediated selection in the divergence of floral traits in heterostylous taxa (Kay and Sargent 2009).

In generalized pollination systems, different pollinator assemblages may lead to spatial variation in pollinator-mediated selection among plant populations (Gómez *et al.* 2009; Harder and Johnson 2009; Chapurlat *et al.* 2015). For instance, *Aquilegia coerulea* populations visited by *Sphinx vashti* have longer spurs than do populations visited by *Hyles lineate* (Sphingidae) (Brunet 2009). In most heterostylous species, reproductive success relies on pollinators. Spatial variation in pollinator communities can also impose differential selective pressures on floral traits (Toräng *et al.* 2008; Vanhoenacker *et al.* 2010; Ågren *et al.* 2013; Vanhoenacker *et al.* 2013). In *Narcissus tazetta*, flower tube length differs between long- and short-tongued insect-dominated populations (Arroyo and Dafni 1995). Spatial variation in pollinator composition contributes to variation in pollen limitation in *Primula farinosa* populations, which results in differential selective pressures on scape length in space (Vanhoenacker *et al.* 2006). Furthermore, spatial variation in pollinator assemblages is expected to lead to the evolutionary breakdown of heterostyly. In *Primula oreodoxa*, the loss of functional pollinators has led to the breakdown of the cross-pollination system in some populations, resulting in evolution from distyly to homostyly (Yuan *et al.* 2017). Therefore, directly quantifying the spatial variation in pollinator-mediated selection on floral traits is helpful to predict the divergence of floral traits among natural populations and its effects.

In heterostylous species, morph-specific variation in selective pressures may also lead to the divergence of floral traits (Nishihiro *et al.* 2000). For example, a morph difference in reproductive success in *Primula sieboldii* leads to selection for a higher stigma in the short-styled morph but not in the long-styled morph (Nishihiro *et al.* 2000). This difference in selection is attributed to the differential corolla tube width between morphs, as the narrow corolla tube of the short-styled morph restricts the path of the pollinator proboscis. Because of the different pollination success between morphs, selection on corolla tube length is stronger in the short-styled morph than in the long-styled morph in *P. vulgaris* and *P. veris* populations (Kálmán *et al.* 2007). In addition, a morph-specific difference in pollination success in *N. tazetta* leads to selection for more concentrated nectar in the long-styled flowers than in the short-styled flowers (Arroyo and Dafni 1995). Furthermore, morph-specific variation in pollinator-mediated selection on floral traits plays important roles in the maintenance and evolution of the heterostylous syndrome (Pérez-Barrales and Arroyo 2010; Simón-Porcar *et al.* 2014; Yuan *et al.* 2017). In *Narcissus papyraceus*, its long-tongued pollinators can successfully pollinate the flowers of all morphs, which may be involved in the maintenance of the stylar polymorphism (Arroyo *et al.* 2002; Simón-Porcar *et al.* 2014). In contrast, the short-tongued pollinators lead to less fertility in the short-styled plants than in the long-styled plants, which may lead to the loss of the stylar polymorphism in *N. papyraceus* (Pérez-Barrales and Arroyo 2010). Similar findings have been reported in Rubiaceae (de Castro and de Oliveira 2002; Massinga *et al.* 2005). In primrose species, morph-specific variation in pollinator-mediated selection on corolla tube length is associated with the evolution of self-fertilization from cross-fertilization (de Vos *et al.* 2014). For example, pollinator-mediated selection for shorter corolla tube length may act to (i) reduce morphological mismatch between morphs, as observed in *P. oreodoxa* (Yuan *et al.* 2017), which might lead to selfing; or (ii) reduce reciprocal herkogamy between morphs, which leads to the breakdown of heterostyly, as observed in *P. vulgaris* and *P. veris* (Kálmán *et al.* 2007).

Intriguingly, the functional pollinators and their foraging behaviours vary among natural *Primula secundiflora* populations. Based on previous studies and our own observations (Zhu *et al.* 2015; Wu and Li 2017), bumblebees and syrphid flies are each the dominant pollinators of individual *P. secundiflora* populations. Bumblebees commonly feed on/collect nectar and pollen during visitation; in contrast, syrphid flies commonly collect pollen. In addition, the floral rewards (pollen and nectar) commonly locate at different positions (nectar at the corolla tube bottom, pollen at a lower or upper position in the corolla tube) in the corolla tube between the long- and short-styled flowers of this primrose species. In addition, morphological traits (especially tongue length) differ between these two pollinator assemblages (own observations, unpublished). These differences may create specific and complex pollinator–plant interactions. Bumblebees and syrphid flies may generate different selective pressures on floral traits. This system is an ideal pollination system to study how complex pollinator–plant interactions drive the divergence of floral traits in space and between morphs in heterostylous species. Pollinators commonly generate selective pressures on two types of floral traits because of their different functional characters. Flowering phenology, plant height, number of flowers, floral rewards and other traits commonly influence the plant’s capability to attract pollinators, which is reflected in the number of visits received (Ashman and Morgan 2004; Grindeland *et al.* 2005; Ågren *et al.* 2006; Sandring and Ågren 2009; Munguía-Rosas *et al.* 2011; Ehrlén 2015). In contrast, specific floral architectures (e.g. corolla tube length, corolla tube width, spur length) commonly influence the morphological compatibility of pollinator and flower, thereby affecting pollination success (Harder and Johnson 2009; Boberg *et al.* 2014; Paudel *et al.* 2016). Consequently, we quantified pollinator-mediated selection on five floral traits (flowering onset, plant height, number of flowers, corolla tube length and corolla tube width) that influenced plant attractiveness to or morphological compatibility with pollinators. In the present study, we experimentally quantified the pollinator-mediated selection on floral traits among four different pollinator assemblage-dominated *P. secundiflora* populations. Specially, we aimed to determine whether pollinator-mediated selection leads to (i) spatial variation in the divergence of floral traits and (ii) inter-morph variation in the divergence of floral traits.

## Materials and Methods

### Study species and populations

*Primula secundiflora* **[see** **Supporting Information—Fig. S1** **]** is a distylous [long-style and short-anther phenotype (L-morph); short-style and long-anther phenotype (S-morph)], self- and intra-morph incompatible perennial herb that is widely distributed in the alpine regions of southwest China. This herb produces leaves in a basal rosette and typically has 3–43 flowers in a single umbel. The stamens originate from the corolla tube. The flowering period is from May to August, and the fruiting period is from August to September.

In 2017, we selected two bumblebee-dominated populations at Potatso National Park (PNP) (PNP 1 and 2 populations) and two syrphid fly-dominated populations at Bigutianchi Scenic Spot (BGTC) (BGTC 1 and 2 populations) in Shangri-La Country, Yunnan Province, southwest China (Table 1).

**Table 1.** Geographic information for the four experimental *Primula secundiflora* populations.

Population	Longitude	Latitude	Altitude (m a.s.l.)
BGTC 1	99°41′13.544″E	27°37′53.209″N	3605
BGTC 2	99°38′12.160″E	27°37′24.250″N	3891
PNP 1	99°59′56.814″E	27°51′31.415″N	3633
PNP 2	99°54′44.785″E	27°47′50.213″N	3406

### Field experiments and trait measurements

We randomly marked 400 individuals (200 L-morph individuals and 200 S-morph individuals) in each population and randomly assigned 200 individuals (100 individuals each of L- and S-morph) to each of two pollination treatments: open pollination treatment (C) and supplemental hand pollination treatment (HP). The four studied populations were visited two times per week through the flowering period. On each visit, all open flowers in the HP treatments were pollinated by hand with cross-pollen (i.e. with S-morph pollen for L-morph flowers and L-morph pollen for S-morph flowers) from other individuals located at least 10 m from the target individual. All flowers received supplemental hand pollination at least once. For the S-morph flowers, we followed the methods of Zhu *et al.* (2015); briefly, we punctured the corolla tube near the stigma and brushed dehiscing anthers across receptive stigmas through the hole using tweezers.

We recorded flowering onset (Julian day, day of the year) for each individual when the first flower opened. At the onset of flowering, we measured the plant height of each individual in the experiment (distance from the ground to the topmost flower to the nearest 0.1 cm). For the first three open flowers of each individual, we measured corolla tube length (distance from the corolla tube entrance to the corolla tube bottom) and corolla tube width (width of the corolla tube entrance) to the nearest 0.01 mm with digital calipers. We recorded the number of flowers for each individual at the end of the flowering period.

To quantify female reproductive success, we recorded the number of fruits at maturation and collected all fruits from each individual to determine the seeds per fruit. For each individual, we estimated total seed production as a proxy of female fitness. We quantified pollen limitation for each population and each morph as 1 − (mean female fitness of open-pollinated individuals/mean female fitness of hand-pollinated individuals). The mean female fitness was calculated as the total seed production per individual. We calculated 95 % confidence intervals (CIs) for the pollen limitation estimates using bootstrapping (1000 iterations; boot-package in R; Canty and Ripley 2013).

### Pollinator observations

In 2017, we conducted pollinator observations for the four populations. In each population, we established two or three 2 × 2 m plots. Each plot contained between 100 and 150 *P. secundiflora* individuals. Pollinators were recorded during a series of 30-min sessions between 0830 and 1830 on three sunny days (e.g. 0830–0900, 0930–1000; 5.5 h of observations for each sunny day, 2 days in June and 1 day in July) at the peak flowering period for each population. We recorded a plant visitor as a pollinator if it touched the sexual organs of the flower during its visit (We only considered visitors contacting the stigma of L-morph flowers and the anthers of S-morph flowers because these organs locate at the upper position in the corolla tube.). For each observation session, we recorded the types, numbers and behaviours of the pollinators. We calculated the mean number of pollinators per plot per hour in this study. We caught the dominant pollinators (syrphid flies in the BGTC 1 and 2 populations and bumblebees in the PNP 1 and 2 populations) and brought them to the laboratory. We measured the body lengths and proboscis lengths of the pollinators to the nearest 0.01 mm with digital calipers **[see****Supporting Information—Fig. S2****]**.

### Statistical analysis

Three-way ANOVA was used to test the differences in the floral traits (flowering onset, plant height, number of flowers, corolla tube length and corolla tube width) and female reproductive performance (fruit production, seeds per fruit and female fitness) among populations, morphs (L-morph vs. S-morph) and pollination treatments (C vs. HP). To improve the normal distributions of the data, flowering onset, plant height, number of flowers, corolla tube length, corolla tube width, fruit production, seeds per fruit and female fitness were log_10_ transformed prior to analysis. One-way ANOVA was used to test the differences in the body and proboscis lengths between the syrphid flies and bumblebees. To improve the normal distributions of the data, body length and proboscis length were log_10_ transformed prior to analysis.

Following the methods of Lande and Arnold (1983), we used multiple regression analysis to estimate the net directional selection and pollinator-mediated selection. In the regression models, we used relative female fitness (individual female fitness/mean female fitness, using the original data) and the standardized traits (with a mean of 0 and a variance of 1, using the original data) as the response variable and explanatory variables, respectively. Due to the significant differences in corolla tube length, corolla tube width and female fitness between the L-morph and S-morph, we estimated relative female fitness and standardized the traits separately for each population, morph and pollination treatment. In addition, we estimated selection gradients separately for each population, morph and pollination treatment. We quantified the directional selection gradients (β_*i*_) using multiple linear regression models. We initially included cross-product terms (γ_*ij*_, between floral traits) to quantify correlational selection. A few correlational selection gradients were statistically significant, and the variance inflation factors (VIFs) exceeded 10, which indicated substantial multicollinearity in these models (Quinn and Keough 2002). Consequently, we did not include the non-linear and cross-product terms in the regression models. To test for multicollinearity in these linear regression models, we calculated the VIFs for the linear terms. All VIFs were <1.7, indicating no multicollinearity problem (Quinn and Keough 2002).

To examine whether net directional selection varied among the populations and between the morphs, we analysed the data from the plants in the open pollination treatment (C) in the four populations in an ANCOVA (Model A; Table 2). In Model A, a significant trait × population term indicated that the net directional selection varied among the populations; in addition, a significant trait × morph term indicated the net directional selection varied between morphs.

**Table 2.** ANCOVA models testing variations in net directional selection and pollinator-mediated selection among the populations and morphs.

Terms (ANCOVA model)	Net directional selection (Model A)	Pollinator-mediated selection (Model B)
Response variable	Relative female fitness	Relative female fitness
	Five standardized traits	Five standardized traits
	Population	Population
	Morph	Morph
	Trait × population	Pollination (C vs. HP)
	Trait × morph	Trait × population
Explanatory variables	Trait × population × morph	Trait × morph
		Trait × pollination
		Trait × population × morph
		Trait × population × pollination
		Trait × morph × pollination
		Trait × population × morph × pollination

To test whether pollinator-mediated selection varied among the populations and between the morphs, we analysed the data from the plants of both the open pollination (C) and supplemental hand pollination (HP) treatments in the four populations in an ANCOVA (Model B; Table 2). In Model B, a significant trait × population × pollination term indicated that the pollinator-mediated selection varied among the populations; in addition, a significant trait × morph × pollination term indicated the pollinator-mediated selection varied between morphs. Because some trait × population × pollination and trait × morph × pollination terms were significant, we further tested the effect of pollination treatment (C vs. HP) on linear selection gradients separately for each population and each morph to determine whether there was significant pollinator-mediated selection. To quantify pollinator-mediated selection, we subtracted the estimated selection gradients of each trait for plants that received supplemental hand pollination (β_HP_) from the estimate obtained for plants under open pollination (β_C_) (Δβ_poll_ = β_C_ − β_HP_) (Chapurlat *et al.* 2015).

All analyses were performed with R 3.3.2 (R Core Team 2017). We used Excel (2007) (Microsoft, LA, USA) and Photoshop CS4 (Adobe Systems, LA, USA) to generate the graphs.

## Results

### Floral traits, female reproductive success and pollen limitation

All five phenotypic traits varied among the populations, and corolla tube length and corolla tube width varied between the morphs (Table 3; **see****Supporting Information—Table S1**). The plants in the BGTC 1 population were taller than the plants in the other populations and had more flowers and an earlier flowering onset date for both the L- and S-morph flowers (Table 3). The plants in the BGTC 2 population had shorter corolla tube lengths and larger corolla tube widths in both the L- and S-morph flowers than did the plants in the other populations (Table 3). Corolla tube length was shorter in the L-morph flowers than in the S-morph flowers in all four populations (Table 3; **see****Supporting Information—Table S1**). In addition, corolla tube width was larger in the L-morph flowers than in the S-morph flowers in all populations.

**Table 3.** The five measured floral traits (mean ± SD) of plants in the open pollination treatment (C) and supplemental hand pollination treatment (HP) in morphs of the four *Primula secundiflora* populations. Sample sizes (*n*) are given.

Traits, by study site	L-morph		S-morph	
	C (*n* = 100/98/88/78)	HP (*n* = 97/90/93/95)	C (*n* = 100/91/96/88)	HP (*n* = 97/91/94/89)
Flowering onset (Julian day)				
BGTC 1	154.3 ± 1.3	154.3 ± 1.6	154.2 ± 1.6	154.1 ± 1.3
BGTC 2	154.7 ± 1.9	157.5 ± 5.0	154.0 ± 2.0	155.1 ± 2.4
PNP 1	158.4 ± 1.8	159.2 ± 1.6	160.2 ± 1.5	157.8 ± 1.9
PNP 2	161.2 ± 1.1	160.6 ± 1.3	161.1 ± 1.3	162.4 ± 2.6
Plant height (cm)				
BGTC 1	44.6 ± 7.3	49.2 ± 7.9	45.6 ± 7.7	44.3 ± 5.9
BGTC 2	28.6 ± 5.4	30.5 ± 5.1	29.0 ± 4.9	29.6 ± 5.6
PNP 1	34.6 ± 5.8	34.3 ± 7.2	33.7 ± 7.0	35.6 ± 6.3
PNP 2	28.0 ± 5.5	27.0 ± 6.1	26.7 ± 6.5	27.5 ± 7.9
Number of flowers				
BGTC 1	18.7 ± 6.3	19.3 ± 7.3	18.5 ± 5.4	20.5 ± 6.1
BGTC 2	8.5 ± 2.5	9.7 ± 4.0	9.2 ± 3.4	8.4 ± 3.0
PNP 1	14.8 ± 5.5	15.3 ± 4.2	15.3 ± 7.1	14.7 ± 4.7
PNP 2	13.9 ± 5.3	13.7 ± 4.8	15.4 ± 6.0	14.1 ± 5.1
Corolla tube length (mm)				
BGTC 1	9.27 ± 0.64	9.55 ± 0.57	10.23 ± 0.65	10.03 ± 0.62
BGTC 2	8.53 ± 1.07	8.40 ± 0.93	9.36 ± 0.69	8.94 ± 0.61
PNP 1	10.04 ± 0.77	10.16 ± 0.60	10.18 ± 0.59	10.02 ± 0.69
PNP 2	9.92 ± 0.58	9.75 ± 0.70	10.14 ± 3.15	9.94 ± 0.58
Corolla tube width (mm)				
BGTC 1	3.69 ± 0.23	3.67 ± 0.30	3.02 ± 0.24	3.05 ± 0.23
BGTC 2	3.81 ± 0.42	3.98 ± 0.43	3.25 ± 0.29	3.06 ± 0.32
PNP 1	3.51 ± 0.32	3.44 ± 0.33	2.82 ± 0.25	2.85 ± 0.27
PNP 2	3.31 ± 0.28	3.24 ± 0.28	2.88 ± 0.30	2.84 ± 0.26

The plants in the PNP 1 and 2 populations exhibited more fruits, more seeds per fruit and higher female fitness under open pollination than did the plants in the BGTC 1 and 2 populations (Table 4; **see****Supporting Information—Table S1**). In the BGTC 1 and 2 populations, the L-morph plants exhibited more fruits, more seeds per fruit and more female fitness under open pollination than did the S-morph plants. In contrast, the differences in reproductive success between the L- and S-morph plants in the PNP 1 and 2 populations were not significant (Table 4). Supplemental hand pollination significantly increased fruit production, seeds per fruit and female fitness (Table 4; **see****Supporting Information—Table S1**). The L-morph plants produced more seeds per fruit than did the S-morph plants except in the BGTC 1 population and exhibited higher female fitness under supplemental hand pollination than did the S-morph plants except in the BGTC 1 and PNP 2 populations.

**Table 4.** Female reproductive performance (mean ± SD) under the open pollination (C) and supplemental hand pollination (HP) treatments in L-morph and S-morph flowers of four *Primula secundiflora* populations. Pollen limitation (PL) and its 95 % confidence intervals (CIs) are given.

Morph	Population	Fruit production		Seeds per fruit		Female fitness		PL	Lower CIs	Upper CIs
		C	HP	C	HP	C	HP			
L-morph	BGTC 1	7.1 ± 3.6	15.4 ± 6.6	75.8 ± 20.9	107.2 ± 19.3	569.1 ± 412.0	1701.2 ± 884.8	0.665	0.607	0.721
	BGTC 2	3.5 ± 1.7	6.9 ± 3.5	52.6 ± 16.5	72.1 ± 15.8	192.5 ± 124.0	512.6 ± 324.8	0.624	0.556	0.687
	PNP 1	9.3 ± 4.3	11.7 ± 5.3	94.8 ± 17.8	110.8 ± 17.5	905.9 ± 480.9	1320.2 ± 695.1	0.314	0.202	0.414
	PNP 2	8.2 ± 3.7	10.3 ± 4.2	101.2 ± 19.2	115.1 ± 19.0	857.6 ± 472.5	1226.8 ± 619.6	0.301	0.177	0.404
S-morph	BGTC 1	7.4 ± 4.3	15.8 ± 5.4	65.1 ± 21.7	112.6 ± 13.6	539.9 ± 453.2	1793.3 ± 691.2	0.699	0.638	0.748
	BGTC 2	2.7 ± 1.5	5.7 ± 2.9	42.2 ± 16.7	63.3 ± 15.4	121.1 ± 103.8	367.3 ± 243.2	0.670	0.593	0.733
	PNP 1	9.2 ± 5.3	10.6 ± 5.1	85.0 ± 17.9	110.1 ± 21.0	821.0 ± 553.2	1198.6 ± 693.3	0.315	0.189	0.434
	PNP 2	8.9 ± 4.7	11.1 ± 4.3	99.6 ± 15.8	106.8 ± 19.3	918.0 ± 574.7	1241.7 ± 642.9	0.261	0.135	0.366

Fruit production, seeds per fruit and female fitness were pollen-limited in all four populations. The pollen limitation of female fitness ranged from 0.261 to 0.699 and varied among populations, as indicated by the significant population × pollination interaction for female fitness (Table 4; **see****Supporting Information—Table S1**). The pollen limitation was higher in the BGTC 1 and 2 populations than in the PNP 1 and 2 populations. Pollen limitation was higher in the S-morph flowers than in the L-morph flowers in all of the studied populations except the PNP 2 population.

### Pollinators

**Figure 1.**
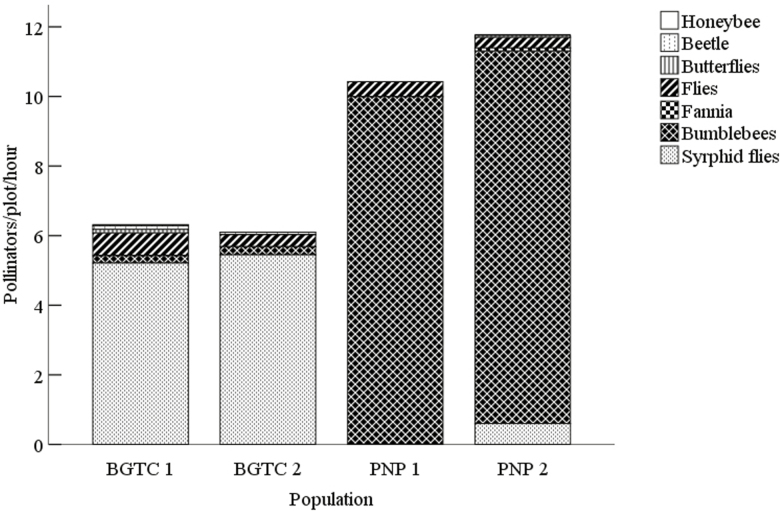
Results of pollinator observations conducted in the four *Primula secundiflora* populations. Bumblebees comprised *Bombus atrocintus*, *B. convexus*, *B. richardsi* and *B. lucorum*.

We conducted 16.5 h of observations at the peak flowering period for each population. In the BGTC 1 and 2 populations, syrphid flies were the dominant pollinators (Fig. 1; **see****Supporting Information—Fig. S1A**), accounting for 82.5 and 89.4 %, respectively, of the total pollinators. The syrphid flies commonly collected pollen during visitation. However, bumblebees were the dominant pollinators in the PNP 1 and 2 populations (Fig. 1; **see****Supporting Information—Fig. S1B and C**), accounting for 95.9 and 91 %, respectively, of the total pollinators. The community of bumblebees included *Bombus atrocintus*, *B. convexus*, *B. richardsi* and *B. lucorum*. *Bombus richardsi* was the most abundant pollinator. Bumblebees commonly fed on/collected nectar and pollen during visitation. In addition, the body lengths (*F*_3, 120_ = 37.096, *P* ＜ 0.001) and proboscis lengths (*F*_3, 120_ = 65.636, *P* ＜ 0.001) were significantly longer for the bumblebees than for the syrphid flies (Table 5).

**Table 5.** Body length and proboscis length (mean ± SD) of syrphid flies and bumblebees in the four experimental *Primula secundiflora* populations. Sample sizes (*n*) are given.

Population	Body length (mm)	Proboscis length (mm)
BGTC 1 (*n* = 25)	14.77 ± 0.22	5.29 ± 0.34
BGTC 2 (*n* = 30)	14.28 ± 0.21	5.35 ± 0.31
PNP 1 (*n* = 35)	17.39 ± 0.28	7.03 ± 0.19
PNP 2 (*n* = 31)	17.67 ± 0.37	7.35 ± 0.18

### Net directional selection

**Figure 2.**
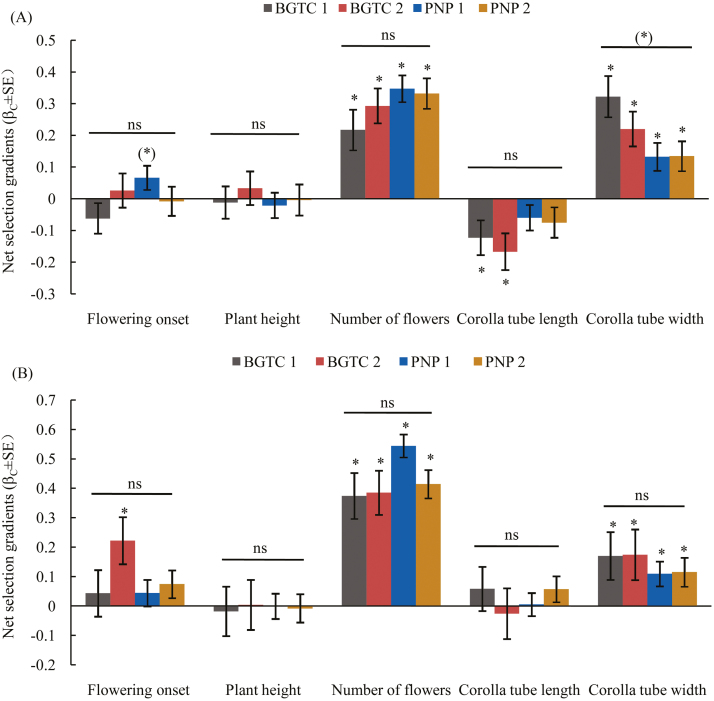
Net directional selection gradients (β_*i*_ ± SE) on flowering onset, plant height, number of flowers, corolla tube length and corolla tube width in the L-morph (A) and S-morph (B) flowers among *Primula secundiflora* populations. Symbols above individual bars indicate the level of significance of the selection gradient. Symbols above the lines indicate whether net directional selection varied among the populations (as indicated by a significant trait × population term in the ANCOVA). *P < 0.05; (*)*P* < 0.1; ^ns^*P* > 0.1.

Net directional selection on flowering onset (*F*_1, 694_ = 4.631, *P* = 0.032), number of flowers (*F*_1, 694_ = 5.837, *P* = 0.016) and corolla tube length (*F*_1, 694_ = 11.378, *P* ＜ 0.001) varied between morphs, as indicated by the significant morph × trait interactions obtained with ANCOVA (Fig. 2; **see****Supporting Information—Table S2**). A later flowering onset date was significantly selected for in the S-morph flowers in the BGTC 2 population (β_C_ = 0.222 ± 0.08; Fig. 2B; **see****Supporting Information—Table S2**); in contrast, selection for a later flowering onset date in the L-morph flowers in the PNP 1 population was marginally significant (Fig. 2A; **see****Supporting Information—Table S2**). There was significant selection for increased number of flowers for both L- and S-morph flowers in all populations (Fig. 2; **see****Supporting Information—Table S2**). A shorter corolla tube length was selected for in the L-morph flowers in the BGTC 1 and 2 populations (β_C_ = −0.123 ± 0.055 and −0.167 ± 0.058, respectively; Fig. 2A; **see****Supporting Information—Table S2**); however, significant net directional selection on this trait was not detected in the S-morph flowers. Significant net directional selection favouring larger corolla tube width in both the L- and S-morph flowers was detected in all populations (Fig. 2; **see****Supporting Information—Table S2**).

### Pollinator-mediated selection

**Figure 3.**
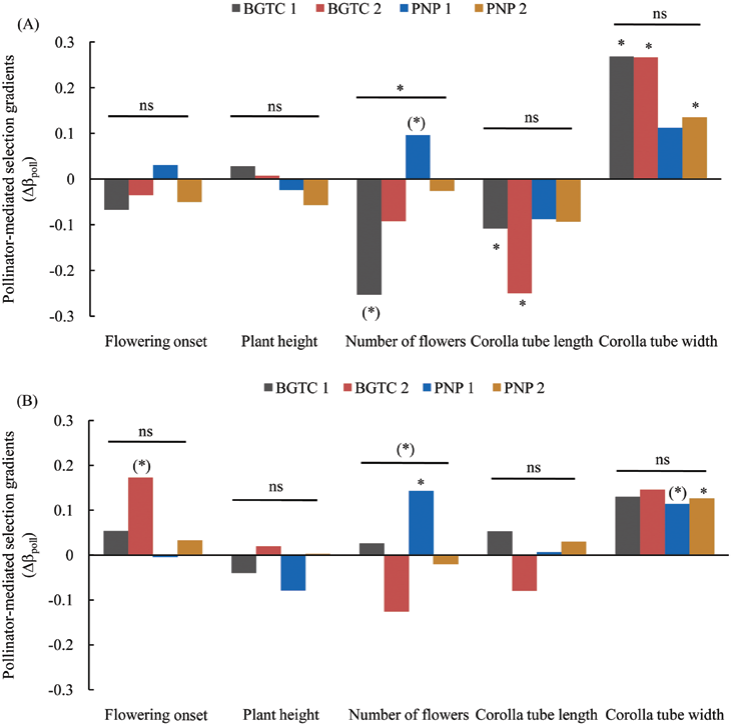
Pollinator-mediated selection gradients on flowering onset, plant height, number of flowers, corolla tube length and corolla tube width in the L-morph (A) and S-morph (B) flowers among *Primula secundiflora* populations. Symbols above individual bars indicate the level of significance of the selection gradient (significant trait × pollination term in the ANCOVA). Symbols above the lines indicate whether pollinator-mediated selection varied among the populations (as indicated by a significant trait × population × pollination term in the ANCOVA). *P < 0.05; (*)*P <* 0.1; ^ns^*P >* 0.1.

Pollinator-mediated selection for later flowering onset was marginally significant in the S-morph flowers of the BGTC 2 population (Δβ_poll_ = 0.173, *P* = 0.071; Fig. 3B; **see****Supporting Information—Fig. S3H**; **Table S2**).

Pollinator-mediated selection on number of flowers varied among the populations (*F*_3, 1399_ = 3.686, *P* = 0.012). In the L-morph flowers, less number of flowers was marginally significantly (*P* = 0.063) selected for in the BGTC 1 population, whereas more number of flowers was marginally significantly (*P* = 0.057) selected for in the PNP 1 population (Fig. 3A; **see****Supporting Information—Fig. S3A and F**; **Table S2**). Pollinator-mediated selection for more number of flowers was only detected in the S-morph flowers of the PNP 1 population (Δβ_poll_ = 0.146, *P* = 0.009; Fig. 3B; **see****Supporting Information—Fig. S3I**; **Table S2**).

Pollinator-mediated selection on corolla tube length varied between morphs, as indicated by the significant corolla tube length × pollination × morph interaction (*F*_1, 1399_ = 7.397, *P* = 0.007). Pollinator-mediated selection for a shorter corolla tube was significant in the L-morph flowers in the BGTC 1 and 2 populations (Δβ_poll_ = −0.108, −0.25; Fig. 3A; **see****Supporting Information—Fig. S3B and D**; **Table S2**). However, pollinator-mediated selection on corolla tube length was not significant in the S-morph flowers.

In the L-morph flowers, pollinator-mediated selection for a larger corolla tube width was detected in all populations (Fig. 3A; **see****Supporting Information—Fig. S3C, E and G**; **Table S2**) except the PNP 1 population (Δβ_poll_ = 0.112, *P* = 0.131). In the S-morph flowers, pollinator-mediated selection for a larger corolla tube width was detected in the PNP 1 (marginally significant, Δβ_poll_ = 0.114, *P* = 0.068) and PNP 2 populations (Δβ_poll_ = 0.126, *P* = 0.022) (Fig. 3B; **see****Supporting Information—Fig. S3J and K**; **Table S2**). Although pollinator-mediated selection on corolla tube width was not significant in the BGTC 1 and 2 populations, it explained most [(0.13/0.17) * 100 % = 76.5 %; (0.146/0.174) * 100 % = 83.9 %] of the net directional selection on this trait (Fig. 3B; **see****Supporting Information—Table S2**).

## Discussion

Bumblebees and syrphid flies were each the most abundant pollinators in different *P. secundiflora* populations. The variation in pollinator assemblage led not only to the difference in reproductive success among the populations but also to differences in reproductive success between the L- and S-morph flowers. This variation in pollinator assemblage contributed to the variation in pollinator-mediated selection on floral traits and might lead to divergence of floral traits in the studied natural primrose populations. In the present study, pollinator-mediated selection on corolla tube length and corolla tube width varied among the populations with different pollinator assemblages and between morphs.

The pattern of differences in the floral traits is consistent with the hypothesis that populations have responded to different selective pressures generated by different pollinators (Pérez-Barrales *et al.* 2007; Gómez *et al.* 2009; Schiestl and Johnson 2013). In the bumblebee-dominated *P. secundiflora* populations, corolla tube width was the target trait under selection. In contrast, both corolla tube length and corolla tube width were target traits under selection in the syrphid fly-dominated populations. There seems to be an association between floral traits and the locally most abundant pollinators; this association may reflect local adaptation, which is a mechanism that results in floral evolution. In *N. papyraceus* and *N. tazetta*, the populations pollinated by long-tongued pollinators show less variation in floral traits than do those pollinated by short-tongued pollinators (Pérez-Barrales *et al.* 2007; Pérez-Barrales *et al.* 2014). Our study yielded similar findings. The bumblebees fed on/collected nectar and pollen, whereas the syrphid flies only collected pollen. In addition, the proboscis length of the bumblebees was longer than that of the syrphid flies. Accordingly, bumblebees generated selective pressures that affected the mean value of single floral traits (corolla tube width), and syrphid flies generated selective pressures that affected the covariation patterns of multiple floral traits (corolla tube length and corolla tube width) in *P. secundiflora*. In daffodils, sexual polymorphisms are associated with the evolution of narrow floral tubes (Barrett and Harder 2005). However, in the present study, bumblebee- and syrphid fly-mediated selection for wider corolla tube in *P. secundiflora* was observed. A wider corolla tube may reduce accurate pollen transfer between morphs and thereby lead to the breakdown of sexual polymorphisms. Alternatively, it can maintain pollination success, especially in the populations pollinated by short-tongued pollinators. In the present study, a wider corolla tube was associated with higher female reproductive success for both morphs, suggesting that selective pressure on this trait is beneficial for the maintenance of sexual polymorphisms in this primrose species.

Intriguingly, morph-specific variation in pollinator-mediated selection on corolla tube length was observed in the syrphid fly-dominated populations but not in the bumblebee-dominated populations. In our studied *Primula* populations, the long-tongued bumblebees sought nectar, which locates at the bottom of the corolla tube; therefore, the bees showed a morphology compatible with that of the flowers. Such bees can easily contact the anthers and stigma in both L- and S-morph flowers, thus promoting pollen transfer between morphs and increasing pollination success. In contrast, syrphid flies commonly collected pollen during visitation, and their proboscis lengths were shorter. In addition, the number of visits per period of syrphid flies was consistently lower than that of bumblebees. The organization of sexual organs (anthers and stigma) differs between the L- and S-morph flowers in *P. secundiflora*. All of these phenomena cause S-morph flowers to be less likely to receive pollen under a syrphid fly-dominated pollination environment and to show lower reproductive success than L-morph flowers. Furthermore, these phenomena have led to morph-specific selective pressures on floral traits, as demonstrated by our results. However, the findings deviate from the predictions in *P. vulgaris* and *P. veris* of stronger selection on corolla tube length in short-styled flowers than in long-styled flowers (Kálmán *et al.* 2007). Our results revealed stronger selection for shorter corolla tube length in the L-morph flowers than the S-morph flowers. Shorter corolla tube length in the L-morph flower may allow the short-tongued pollinator to more easily contact the anthers and may help maintain reproductive success. In contrast, pollinator-mediated selection for shorter corolla tube length reduces the distance between stigma and anther and may affect the reciprocal herkogamy and morphological match between morphs (Kálmán *et al.* 2007; de Vos *et al.* 2014), thus reducing pollination efficiency and reproductive success (Ferrero *et al.* 2011). Furthermore, it may lead to the breakdown of heterostyly. The benefits and costs of selection for shorter corolla tube length imply that morph-specific variation in pollinator-mediated selection on this trait may not persist in this primrose species.

Floral traits that influence the attractiveness to pollinators, morphological compatibility or pollination efficiency may be under pollinator-mediated selection (Alexandersson and Johnson 2002; Harder and Johnson 2009; Ida and Kudo 2010; Boberg *et al.* 2014). In the present study, the traits of corolla tube length and corolla tube width were selected because of their mechanical fit with the functional pollinators. Similar selective pressures have been demonstrated in other heterostylous species, such as *Narcissus triandrus* and *N. tazetta* (Arroyo and Dafni 1995; Hodgins and Barrett 2008). In contrast, pollinators did not generate selective pressures on flowering onset, plant height and number of flowers, traits that might affect plant attractiveness to pollinators. This finding may indicate that these three floral traits do not differently affect bumblebees and syrphid flies. The results of the supplemental hand pollination treatment indicated that most of the selection on these traits could be attributed to non-pollination agents. In many systems, direct relationships exist among flowering onset, plant height, number of flowers and reproductive success. Thus, selection on these traits can be expected independent of pollinator-mediated selection (Jakobsson *et al.* 2009; Parachnowitsch and Kessler 2010; Weber and Kolb 2013; Sletvold *et al.* 2017).

Our estimates of pollinator-mediated selection on floral traits only consider the association between floral traits and female fitness. Variance in fitness is higher for male function than for female function (Arnold and Wade 1984; Benitez-Vieyra *et al.* 2006), which leads to stronger selection through male fitness. For example, in *N. triandrus*, selection for number of flowers is only detected through female function, whereas selection for corolla width and floral tube length are detected through male function (Hodgins and Barrett 2008). More extensive pollinator observations and measures of pollen export from captured pollinators can be expected to provide additional information about the relationships between floral traits and male fitness. In addition, microsatellite markers would be helpful to clearly estimate male function in natural populations. Furthermore, pollinator-mediated selection is influenced by trait heritability and environmental variation (Mitchell-Olds and Rutledge 1986; Rausher 1992; Mauricio and Mojonniner 1997; Totland 2001; Zu *et al.* 2016). At present, it is unknown whether the observed variation in floral traits in *P. secundiflora* is heritable; there is no relevant information available from other *Primula* species. To address these issues, common garden experiments are needed in the future.

Knowledge of the causes of variation in selection on floral traits is key to understanding the processes and patterns of adaptive evolution (MacColl 2011). The specific floral architectures of heterostylous species create complex pollinator–plant interactions that lead to specific and complex pollinator-mediated selective pressures on floral traits. The results of the present study indicate that variation in pollinator assemblages strongly contributes to differences in reproductive success among populations and between morphs in primrose species. This variation can lead to spatial variation in pollinator-mediated selection on floral traits (corolla tube length and corolla tube width) and to inter-morph variation in pollinator-mediated selection on floral traits (corolla tube length). The findings highlight the potential strengths of different pollinator agents in driving floral evolution in this primrose species. They also support previous studies demonstrating the pollinator-driven diversification of floral traits.

## Sources of Funding

This research was supported by the Joint Funds of the National Natural Science Foundation of China and Yunnan Provincial Government (no. U1202261).

## Contributions by the Authors

Y.W. and Q.-J.L. designed the experiment; Y.W. and T.Z. conducted the experiment; Y.W., Z.-Q.Z. and Q.-J.L. conducted the data analysis and manuscript preparation.

## Conflict of Interest

None declared.

## Supporting Information

The following additional information is available in the online version of this article—

**Table S1**. The effect of population, morph (L-morph vs. S-morph) and pollination (C vs. HP) on floral traits and female reproductive success analysed with three-way ANOVA.

**Table S2.** Linear selection gradients (β_*i*_ ± SE) and associated *P*-values among open-pollinated control plants (C) and hand-pollinated plants (HP) in the four *Primula secundiflora* populations. Pollinator-mediated selection (Δβ_poll_ = β_C_ − β_HP_) and *P*-values association with the trait × pollination treatment interactions in ANCOVAs conducted separately for each morph and each population are also given.

**Figure S1**. *Primula secundiflora* and its dominant pollinators in the studied populations.

**Figure S2**. Illustration of the morphological traits measured in this study on dominant pollinators, syrphid fly (A) and bumblebee (B).

**Figure S3**. Standardized linear phenotypic selection gradients for flowering onset, number of flowers, corolla tube length and corolla tube width in open pollination plants (C, open circles, solid line) and in supplemental hand pollination plants (HP, closed circles, dashed line) at BGTC 1 (A, B, C), BGTC 2 (D, E, H), PNP 1 (F, I, J) and PNP 2 populations (G, K).

**Supporting Data**. An excel file of the data from the present study can be found in the online version of this article.

Supplementary Information

Supplementary Data
