# Supplementary material for: Pollinator-mediated selection on floral traits varies in space and between morphs in Primula secundiflora
Source: AoB Plants. 2018 Oct 1;10(5):ply059. doi: 10.1093/aobpla/ply059 (PMC6205359; doi:10.1093/aobpla/ply059)
Supplement: Supplementary Information [file ply059_suppl_supplementary_information.doc]

**Supplementary Information**

**Table S1.** The effect of population, morph (L-morph vs. S-morph) and pollination (C vs. HP) on floral traits and female reproductive success analysed with three-way ANOVA.

**Table S2.** Linear selection gradients (β_i_ ± SE) and associated *P*-values among open-pollinated control plants (C) and hand-pollinated plants (HP) in the four *P. secundiflora* populations. Pollinator-mediated selection (Δβ_poll_ = β_C_ – β_HP_) and *P*-values association with the trait × pollination treatment interactions in ANCOVAs conducted separately for each morph and each population are also given.

**Figure S1.** *Primula secundiflora* and its dominant pollinators in the studied populations.

**Figure S2.** Illustration of the morphological traits measured in this study on dominant pollinators, syrphid fly (**A**) and bumblebee (**B**).

**Figure S3.** Standardized linear phenotypic selection gradients for flowering onset, number of flowers, corolla tube length and corolla tube width in open pollination plants (C, open circles, solid line) and in supplemental hand pollination plants (HP, closed circles, dashed line) at BGTC 1 (**A, B, C**), BGTC 2 (**D, E, H**), PNP 1 (**F, I, J**) and PNP 2 populations (**G, K**).

**Table S1.** The effect of population, morph (L-morph vs. S-morph) and pollination (C vs. HP) on floral traits and female reproductive success analysed with three-way ANOVA. Bold *P*-values indicate significant effects (at the significance level of 0.05).

| Traits and reproductive success | Population | |  | Morph | |  | Pollination | |  | Population × Morph | |  | Population × Pollination | |  | Morph × Pollination | |  | Population × Morph × Pollination | |
| --- | --- | --- | --- | --- | --- | --- | --- | --- | --- | --- | --- | --- | --- | --- | --- | --- | --- | --- | --- | --- |
|  | *F*_3,1484_ | *P* |  | *F*_1,1484_ | *P* |  | *F*_1,1484_ | *P* |  | *F_3_*_,1484_ | *P* |  | *F*_3,1484_ | *P* |  | *F*_1,1484_ | *P* |  | *F*_3,1484_ | *P* |
| Flowering onset | 920.804 | **＜0.001** |  | 2.341 | 0.126 |  | 12.925 | **＜0.001** |  | 20.481 | **＜0.001** |  | 29.004 | **＜0.001** |  | 13.712 | **＜0.001** |  | 24.759 | **＜0.001** |
| Plant height | 539.384 | **＜0.001** |  | 2.786 | 0.095 |  | 4.896 | **0.027** |  | .926 | 0.428 |  | 1.318 | 0.267 |  | .184 | 0.668 |  | 5.498 | **0.001** |
| Number of flowers | 285.716 | **＜0.001** |  | .193 | 0.660 |  | .301 | 0.583 |  | 1.621 | 0.183 |  | 1.274 | 0.282 |  | 1.844 | 0.175 |  | 2.266 | 0.079 |
| Corolla tube length | 190.309 | **＜0.001** |  | 86.084 | **＜0.001** |  | 3.944 | **0.047** |  | 21.723 | **＜0.001** |  | 2.754 | **0.041** |  | 6.933 | **0.009** |  | 2.294 | 0.076 |
| Corolla tube width | 143.984 | **＜0.001** |  | 1374.814 | **＜0.001** |  | 1.931 | 0.165 |  | 10.663 | **＜0.001** |  | .681 | 0.564 |  | 1.199 | 0.274 |  | 9.791 | **＜0.001** |
| Fruit production | 253.327 | **＜0.001** |  | 5.304 | **0.021** |  | 356.877 | **＜0.001** |  | 6.947 | **＜0.001** |  | 33.783 | **＜0.001** |  | .663 | 0.416 |  | .243 | 0.866 |
| Seeds per fruit | 512.817 | **＜0.001** |  | 48.165 | **＜0.001** |  | 523.806 | **＜0.001** |  | 6.608 | **＜0.001** |  | 44.919 | **＜0.001** |  | 13.319 | **＜0.001** |  | 5.843 | **0.001** |
| Female fitness | 437.879 | **＜0.001** |  | 20.080 | **＜0.001** |  | 564.712 | **＜0.001** |  | 9.097 | **＜0.001** |  | 50.026 | **＜0.001** |  | 4.165 | **0.041** |  | .950 | 0.416 |

**Table S2.** Linear selection gradients (β_i_ ± SE) and associated *P*-values among open-pollinated control plants (C) and hand-pollinated plants (HP) in the four *P. secundiflora* populations. Pollinator-mediated selection (Δβ_poll_ = β_C_ – β_HP_) and *P*-values association with the trait × pollination treatment interactions in ANCOVAs conducted separately for each morph and each population are also given. Significant selection estimates and their *P*-values are indicated in bold.

| Morph | Traits, by site | C | |  | HP | |  | Pollinator-mediated selection | |
| --- | --- | --- | --- | --- | --- | --- | --- | --- | --- |
|  |  | β_i_ ± SE | *P* |  | β_i_ ± SE | *P* |  | Δβ_poll_ | *P* |
| L-morph | BGTC 1 |  |  |  |  |  |  |  |  |
|  | Flowering onset | -0.062±0.048 | 0.198 |  | 0.005±0.023 | 0.816 |  | -0.067 | 0.147 |
|  | Plant height | -0.012±0.051 | 0.812 |  | -0.040±0.022 | 0.072 |  | 0.028 | 0.632 |
|  | Number of flowers | **0.217±0.064** | **0.001** |  | **0.470±0.022** | **＜0.001** |  | -0.253 | 0.063 |
|  | Corolla tube length | **-0.123±0.055** | **0.028** |  | -0.015±0.022 | 0.485 |  | **-0.108** | **0.004** |
|  | Corolla tube width | **0.322±0.065** | **＜0.001** |  | **0.054±0.022** | **0.016** |  | **0.268** | **＜0.001** |
|  | BGTC 2 |  |  |  |  |  |  |  |  |
|  | Flowering onset | 0.026±0.054 | 0.627 |  | 0.061±0.059 | 0.300 |  | -0.035 | 0.883 |
|  | Plant height | 0.033±0.053 | 0.538 |  | 0.026±0.055 | 0.619 |  | 0.007 | 0.998 |
|  | Number of flowers | **0.293±0.055** | **＜0.001** |  | **0.385±0.055** | **＜0.001** |  | -0.092 | 0.805 |
|  | Corolla tube length | **-0.167±0.058** | **0.005** |  | 0.083±0.060 | 0.168 |  | **-0.25** | **0.042** |
|  | Corolla tube width | **0.220±0.055** | **＜0.001** |  | -0.046±0.062 | 0.463 |  | **0.266** | **0.001** |
|  | PNP 1 |  |  |  |  |  |  |  |  |
|  | Flowering onset | 0.066±0.038 | 0.089 |  | 0.036±0.058 | 0.540 |  | 0.03 | 0.858 |
|  | Plant height | -0.021±0.040 | 0.606 |  | 0.003±0.056 | 0.962 |  | -0.024 | 0.998 |
|  | Number of flowers | **0.347±0.042** | **＜0.001** |  | **0.251±0.051** | **＜0.001** |  | **0.096** | **0.057** |
|  | Corolla tube length | -0.060±0.040 | 0.137 |  | 0.028±0.052 | 0.599 |  | -0.088 | 0.289 |
|  | Corolla tube width | **0.132±0.044** | **0.004** |  | 0.020±0.056 | 0.723 |  | 0.112 | 0.131 |
|  | PNP 2 |  |  |  |  |  |  |  |  |
|  | Flowering onset | -0.008±0.046 | 0.868 |  | 0.042±0.040 | 0.297 |  | -0.05 | 0.458 |
|  | Plant height | -0.004±0.049 | 0.940 |  | 0.053±0.041 | 0.195 |  | -0.057 | 0.309 |
|  | Number of flowers | **0.332±0.048** | **＜0.001** |  | **0.358±0.038** | **＜0.001** |  | -0.026 | 0.622 |
|  | Corolla tube length | -0.075±0.048 | 0.12 |  | 0.018±0.036 | 0.620 |  | -0.093 | 0.107 |
|  | Corolla tube width | **0.134±0.047** | **0.006** |  | -0.001±0.037 | 0.980 |  | **0.135** | **0.047** |
| S-morph | BGTC 1 |  |  |  |  |  |  |  |  |
|  | Flowering onset | 0.043±0.079 | 0.585 |  | -0.011±0.015 | 0.476 |  | 0.054 | 0.535 |
|  | Plant height | -0.018±0.084 | 0.826 |  | 0.022±0.017 | 0.191 |  | -0.04 | 0.907 |
|  | Number of flowers | **0.374±0.078** | **＜0.001** |  | **0.348±0.016** | **＜0.001** |  | 0.026 | 0.627 |
|  | Corolla tube length | 0.058±0.075 | 0.441 |  | 0.005±0.016 | 0.755 |  | 0.053 | 0.323 |
|  | Corolla tube width | **0.170±0.081** | **0.038** |  | **0.040±0.015** | **0.008** |  | 0.13 | 0.112 |
|  | BGTC 2 |  |  |  |  |  |  |  |  |
|  | Flowering onset | **0.222±0.080** | **0.006** |  | 0.049±0.044 | 0.269 |  | 0.173 | 0.071 |
|  | Plant height | 0.004±0.085 | 0.965 |  | -0.016±0.045 | 0.718 |  | 0.02 | 0.885 |
|  | Number of flowers | **0.385±0.075** | **＜0.001** |  | **0.511±0.047** | **＜0.001** |  | -0.126 | 0.187 |
|  | Corolla tube length | -0.026±0.086 | 0.760 |  | 0.054±0.045 | 0.226 |  | -0.08 | 0.598 |
|  | Corolla tube width | **0.174±0.086** | **0.046** |  | 0.028±0.044 | 0.527 |  | 0.146 | 0.121 |
|  | PNP 1 |  |  |  |  |  |  |  |  |
|  | Flowering onset | 0.044±0.045 | 0.330 |  | 0.048±0.046 | 0.297 |  | -0.004 | 0.725 |
|  | Plant height | -0.001±0.043 | 0.988 |  | 0.078±0.048 | 0.106 |  | -0.079 | 0.779 |
|  | Number of flowers | **0.544±0.039** | **＜0.001** |  | **0.401±0.044** | **＜0.001** |  | **0.143** | **0.009** |
|  | Corolla tube length | 0.005±0.039 | 0.905 |  | -0.002±0.047 | 0.962 |  | 0.007 | 0.495 |
|  | Corolla tube width | **0.109±0.042** | **0.010** |  | -0.005±0.046 | 0.921 |  | 0.114 | 0.068 |
|  | PNP 2 |  |  |  |  |  |  |  |  |
|  | Flowering onset | 0.074±0.047 | 0.117 |  | 0.041±0.026 | 0.113 |  | 0.033 | 0.352 |
|  | Plant height | -0.008±0.048 | 0.869 |  | -0.011±0.027 | 0.680 |  | 0.003 | 0.784 |
|  | Number of flowers | **0.414±0.048** | **＜0.001** |  | **0.434±0.028** | **＜0.001** |  | -0.020 | 0.640 |
|  | Corolla tube length | 0.057±0.044 | 0.195 |  | 0.027±0.026 | 0.315 |  | 0.030 | 0.278 |
|  | Corolla tube width | **0.115±0.049** | **0.022** |  | -0.011±0.027 | 0.676 |  | **0.126** | **0.022** |


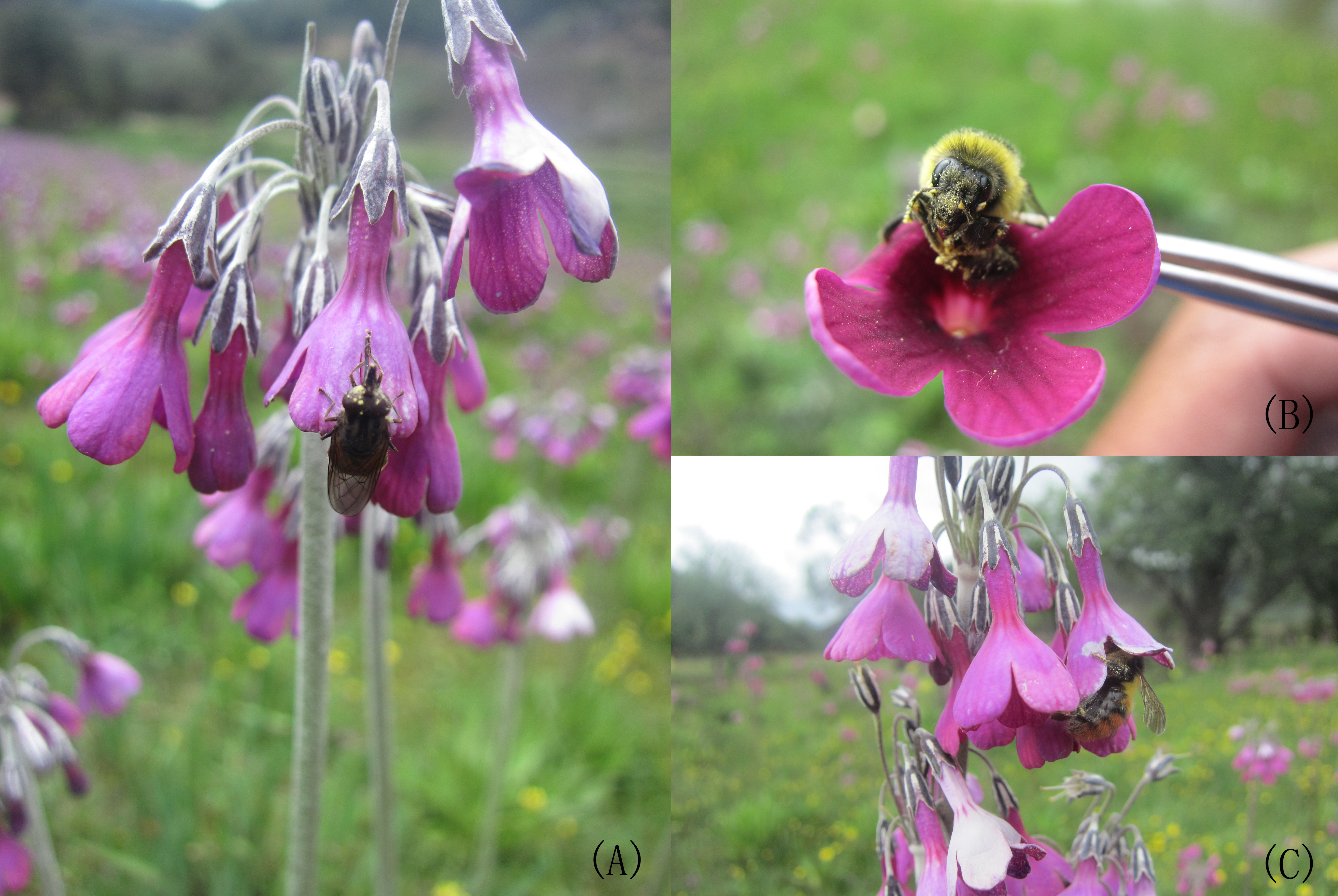


**Figure S1.** *Primula secundiflora* and its dominant pollinators in the studied populations. (**A**), syrphid fly; (**B, C**), bumblebee.


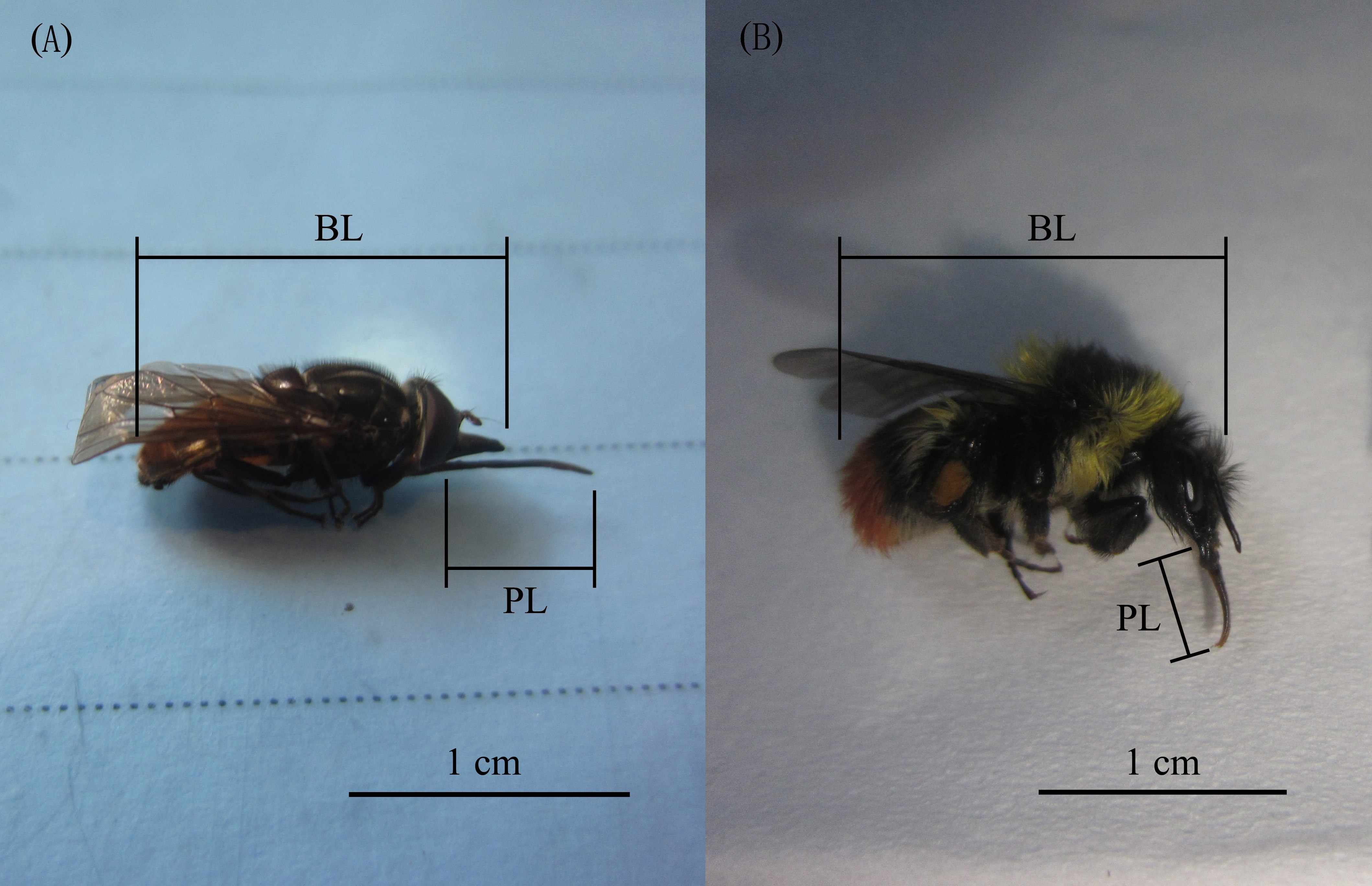


**Figure S2.** Illustration of the morphological traits measured in this study on dominant pollinators, syrphid fly (**A**) and bumblebee (**B**). BL, body length; PL, proboscis length.


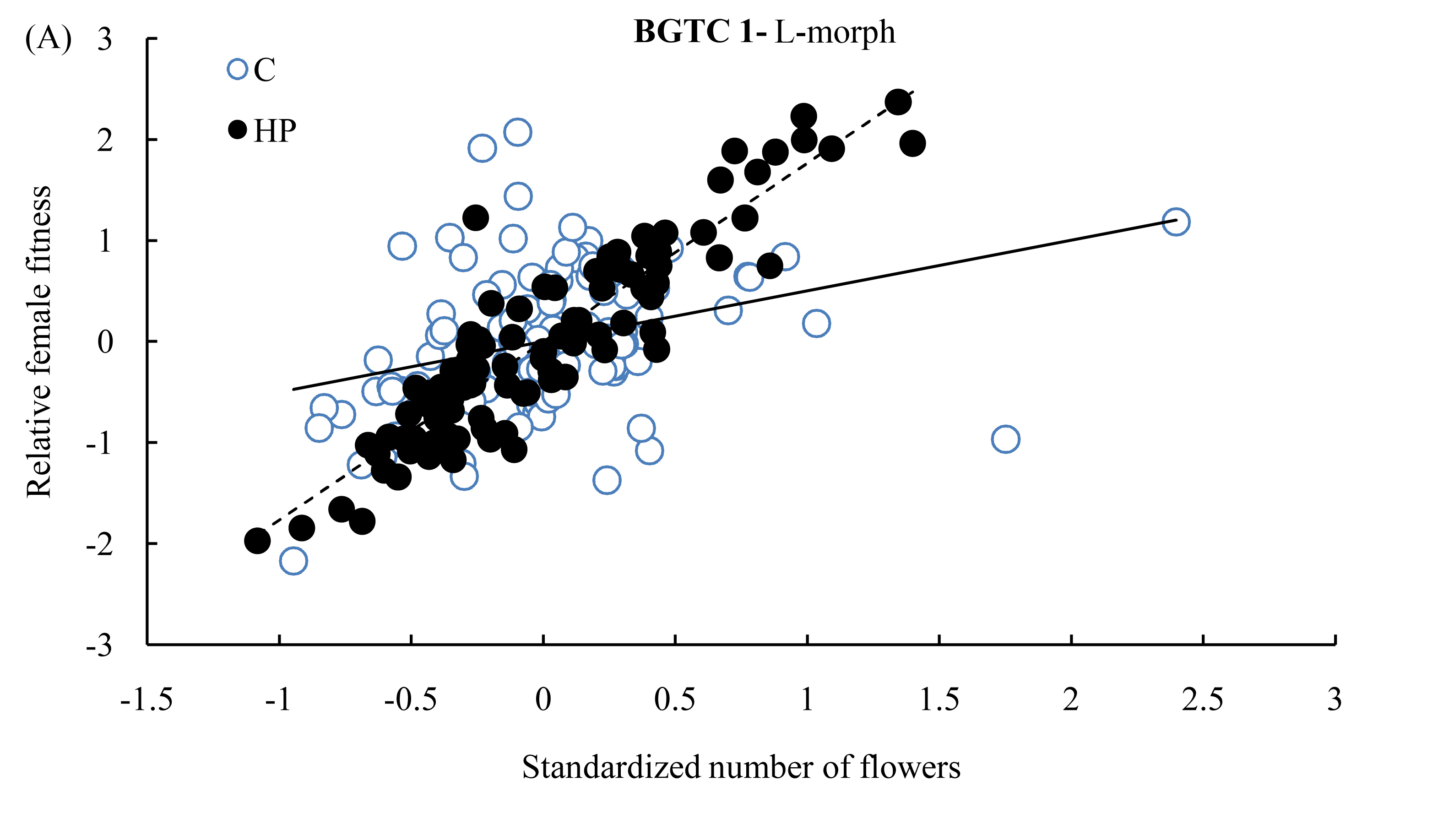


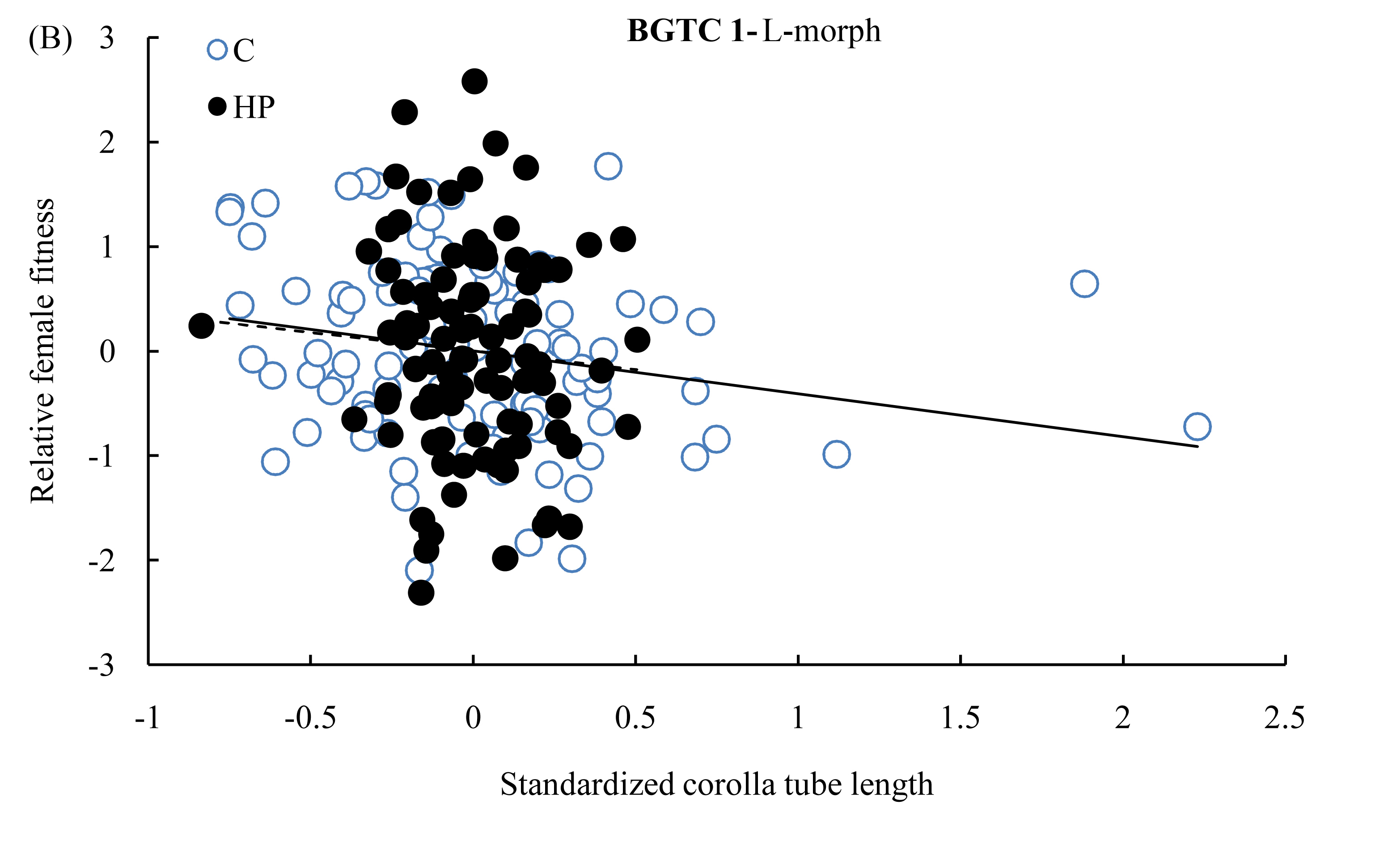


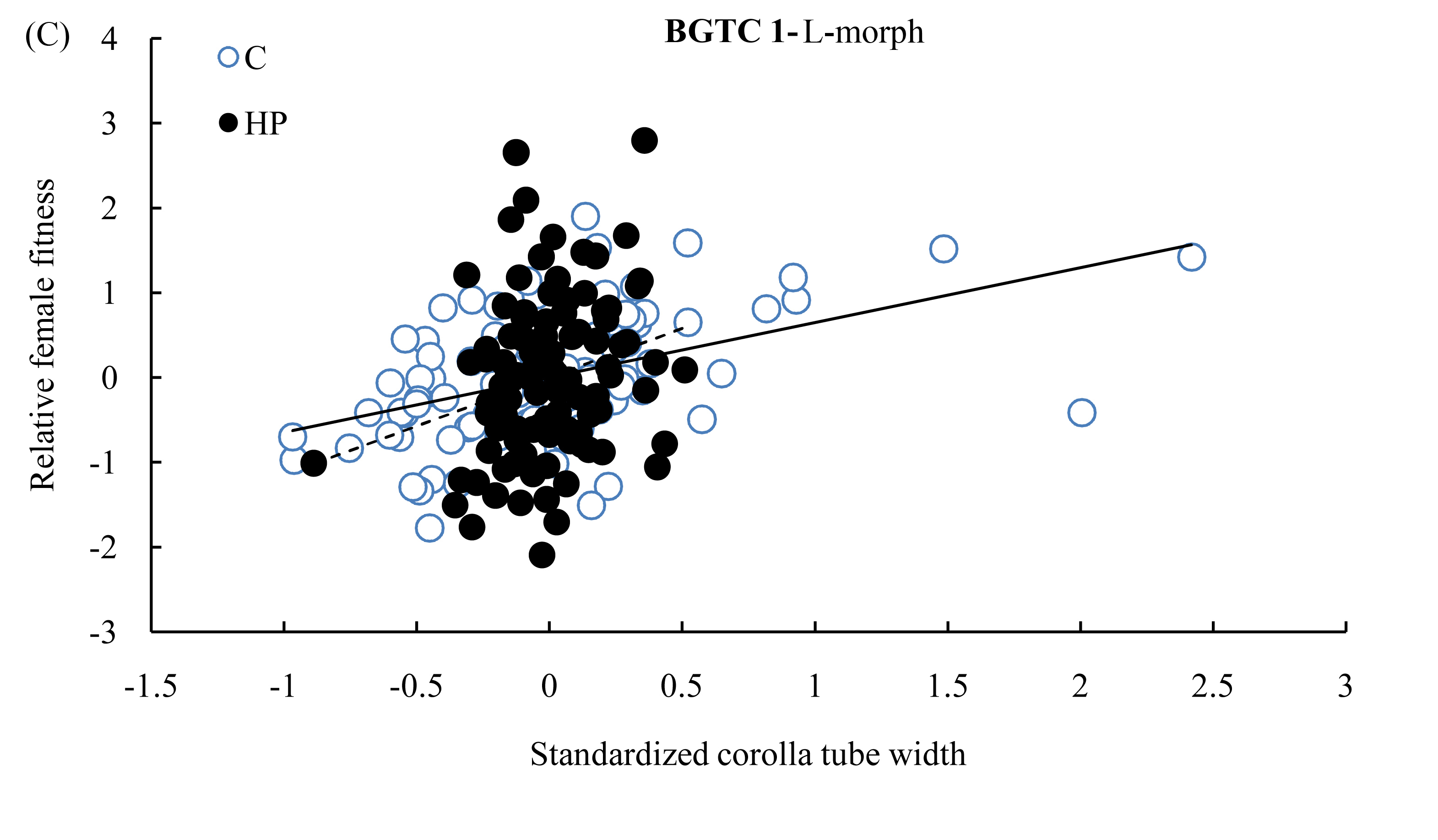


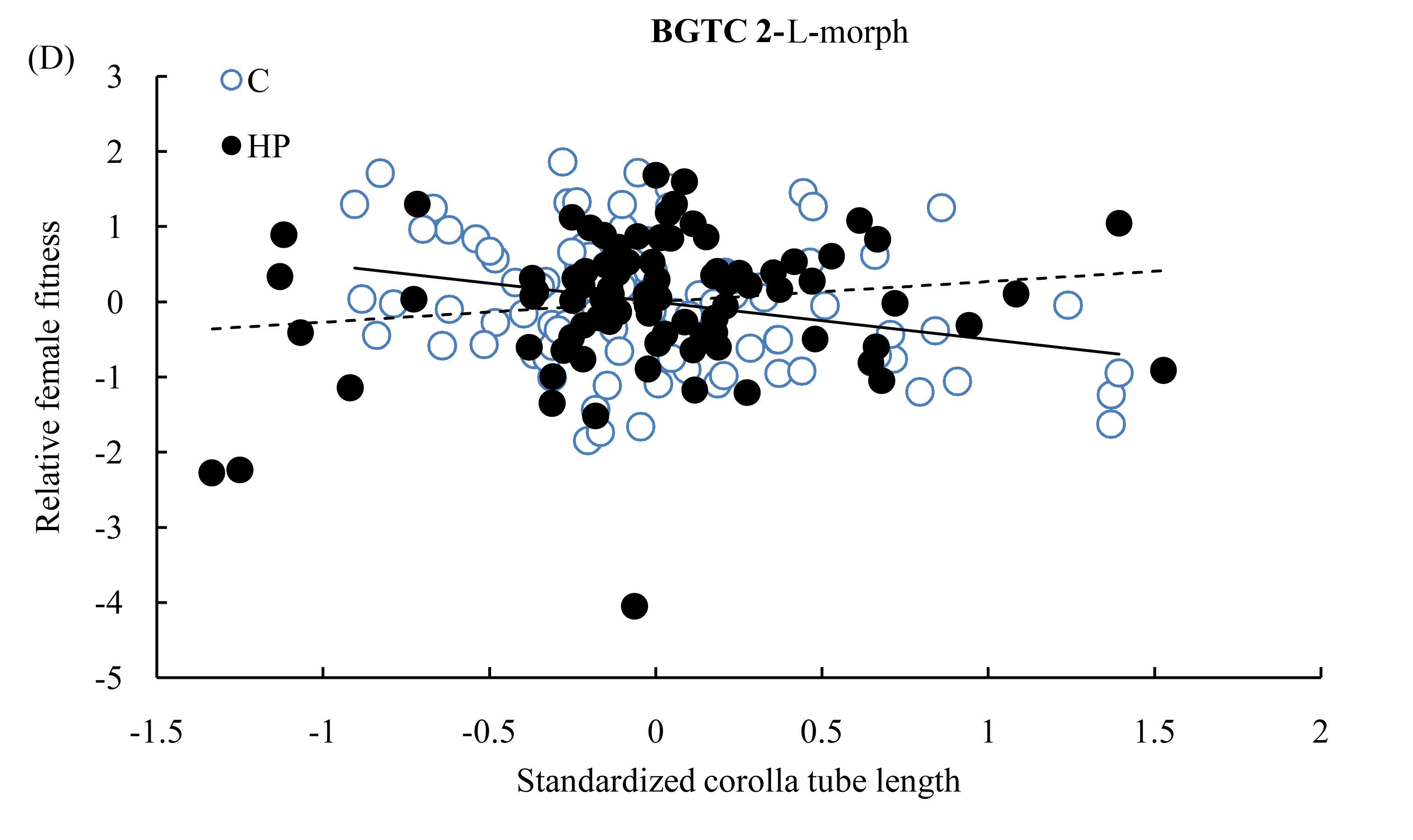


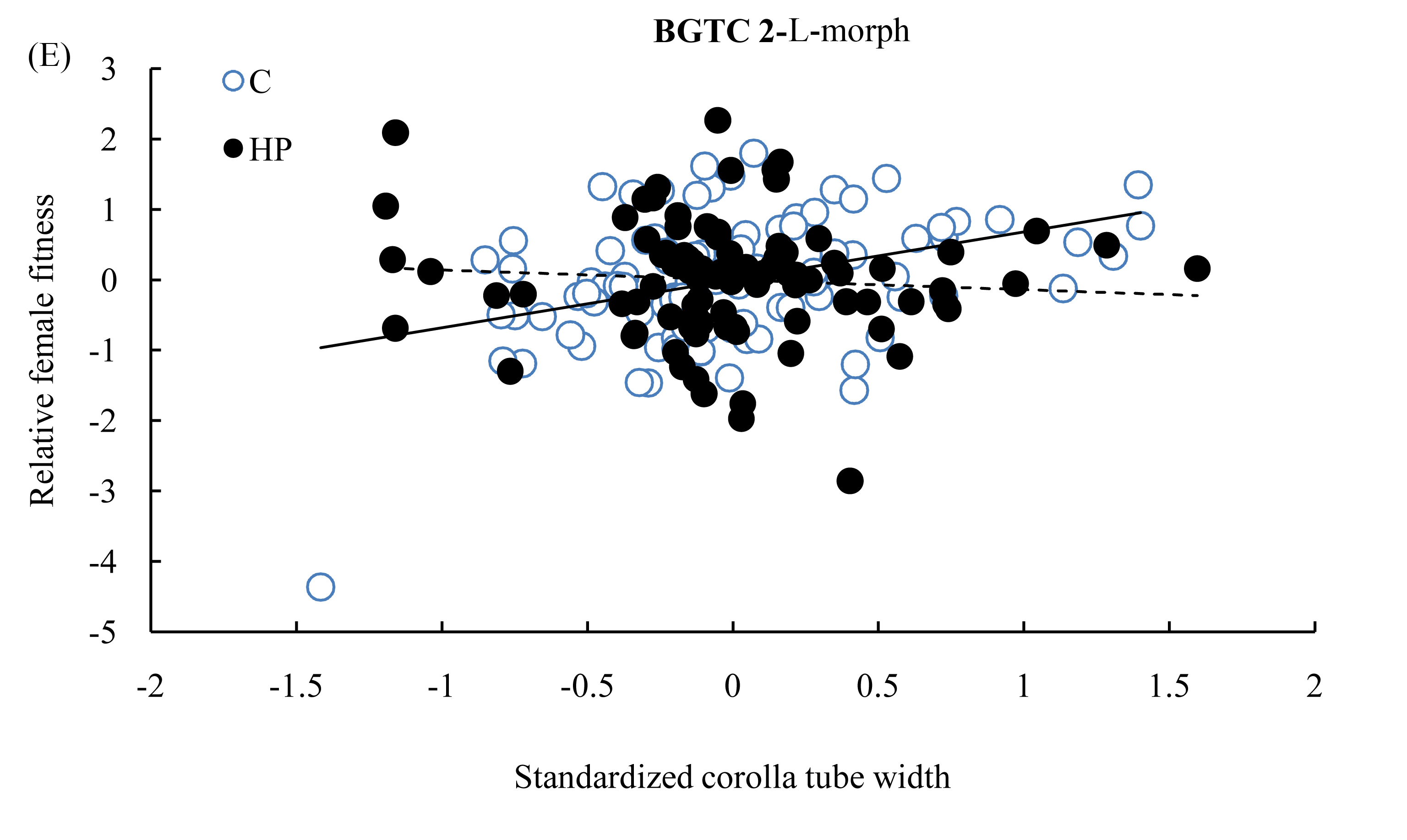


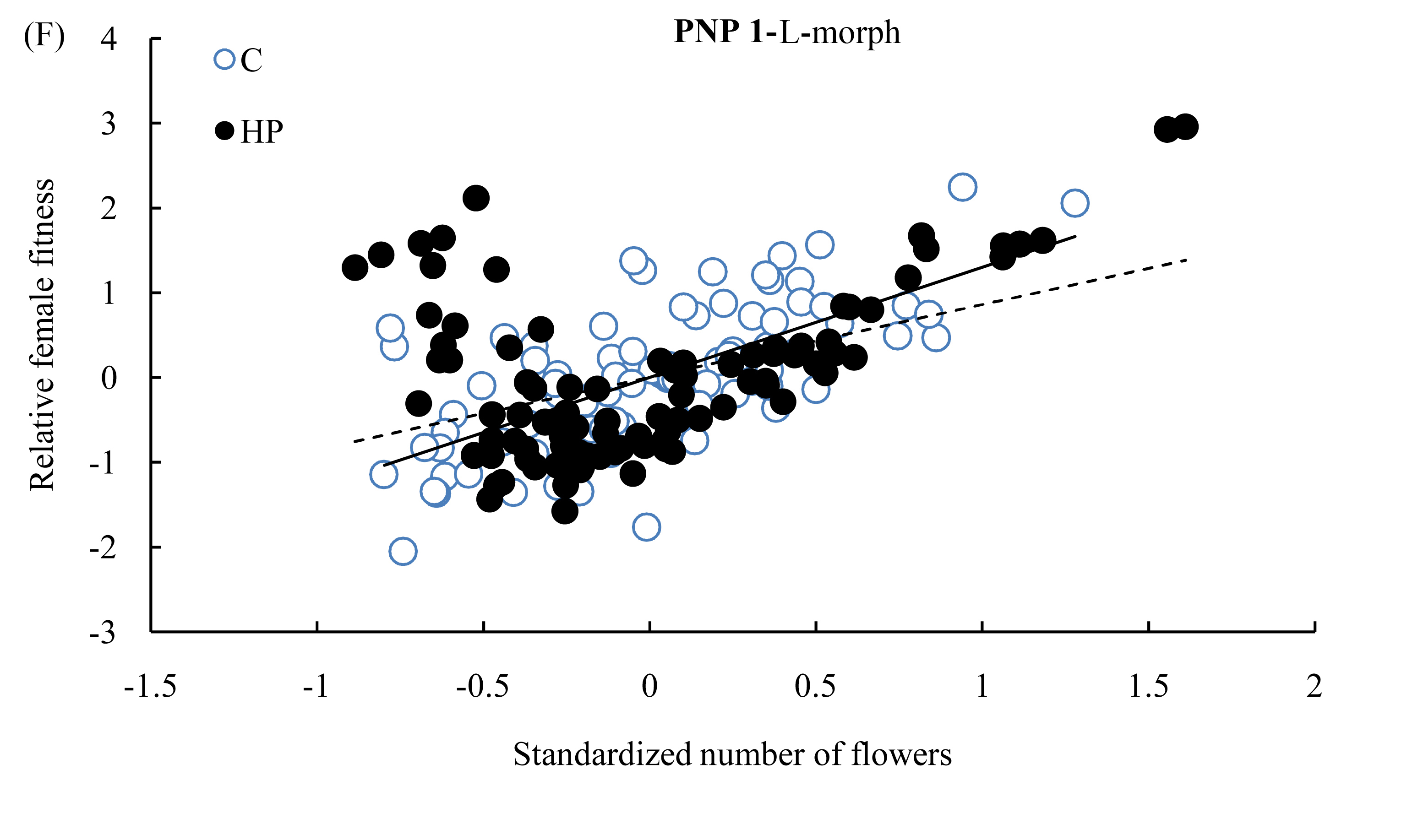


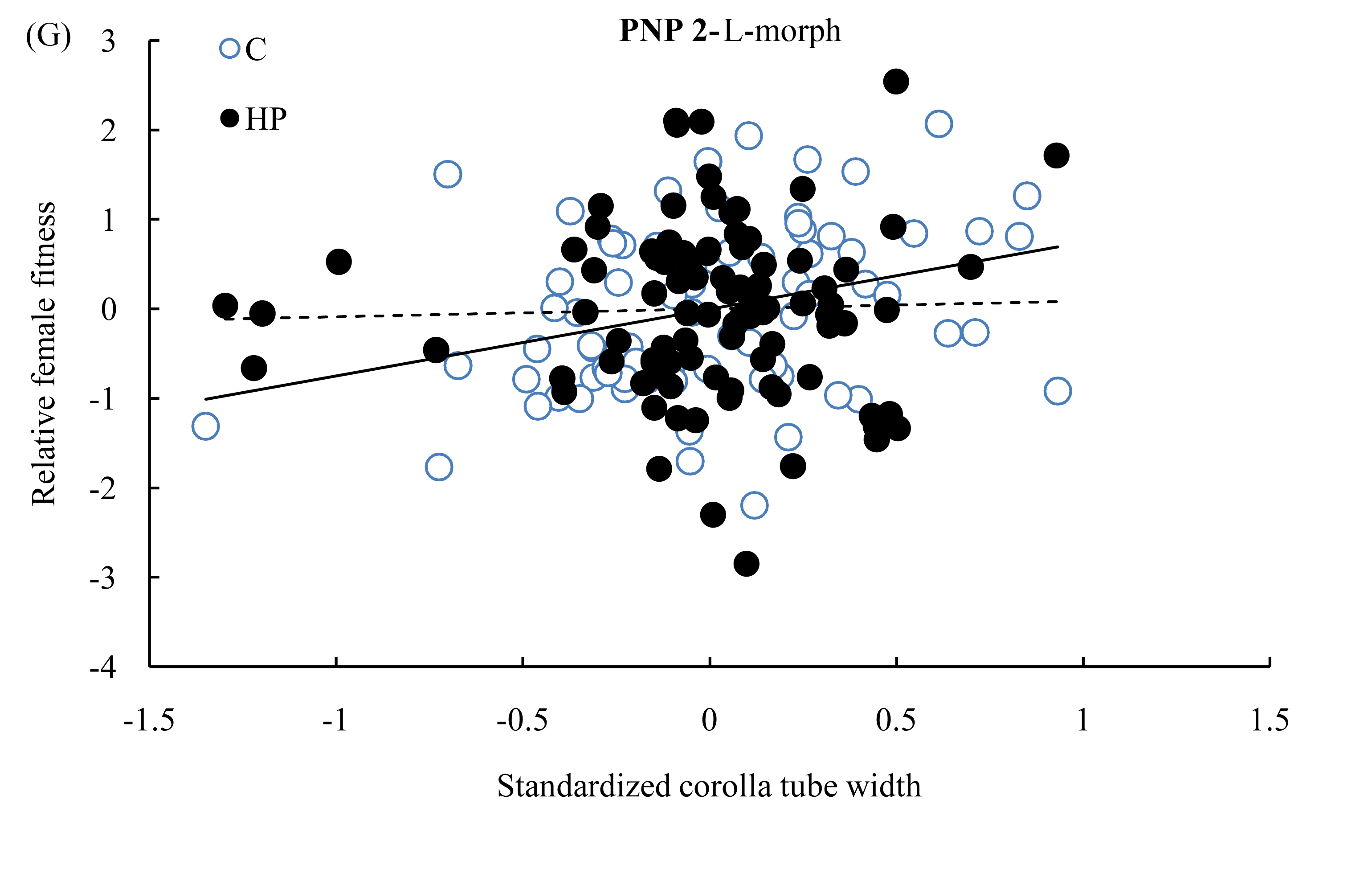


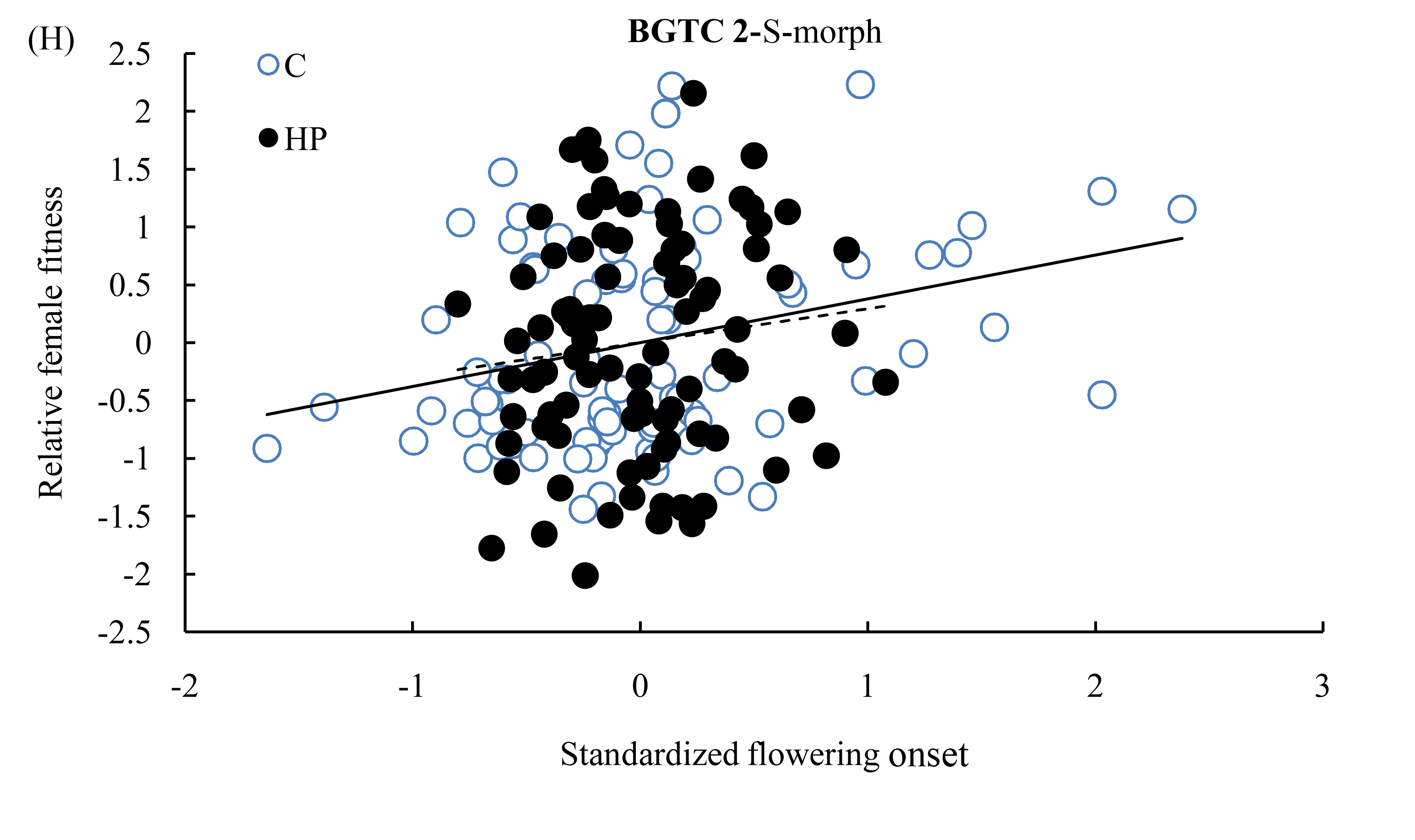


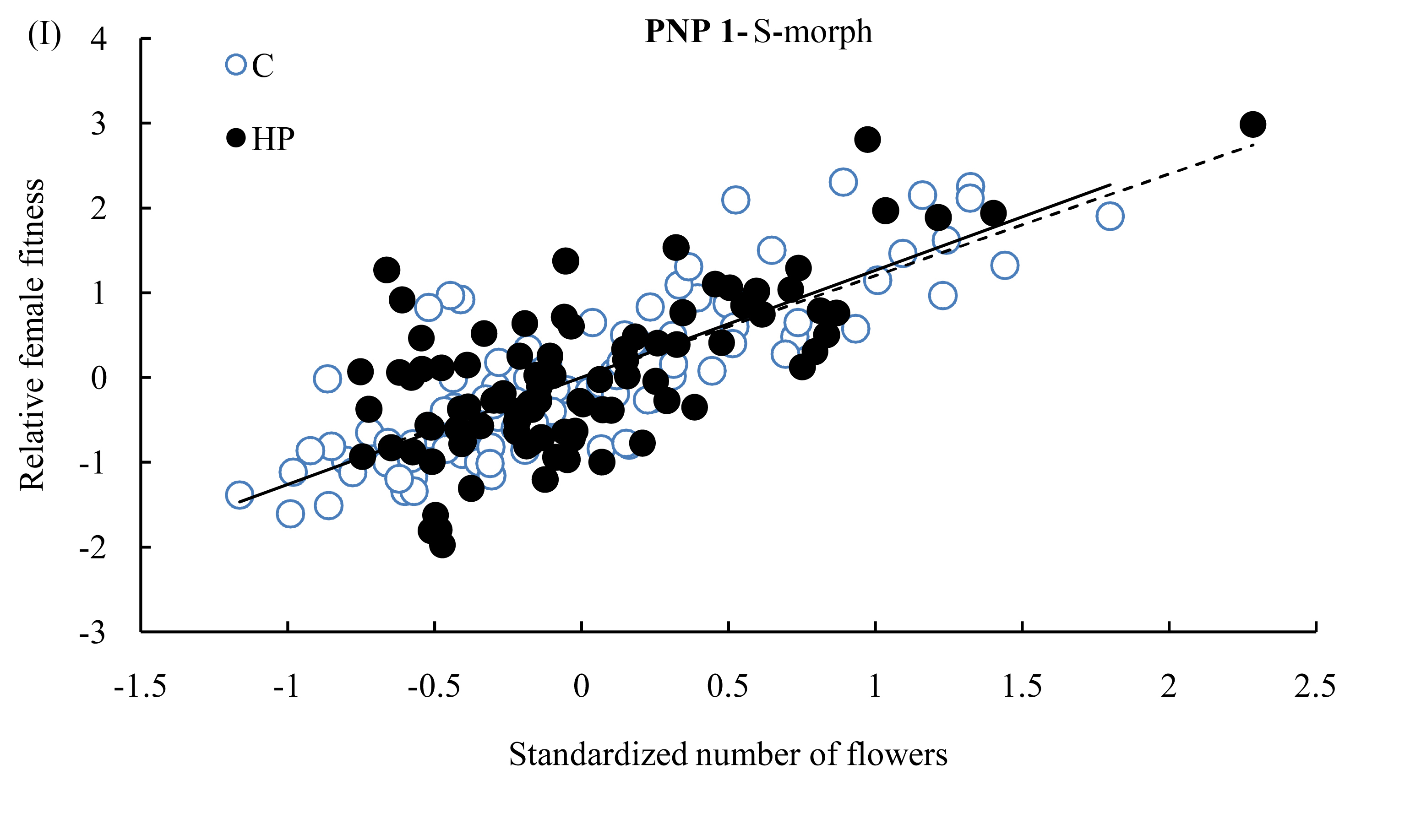


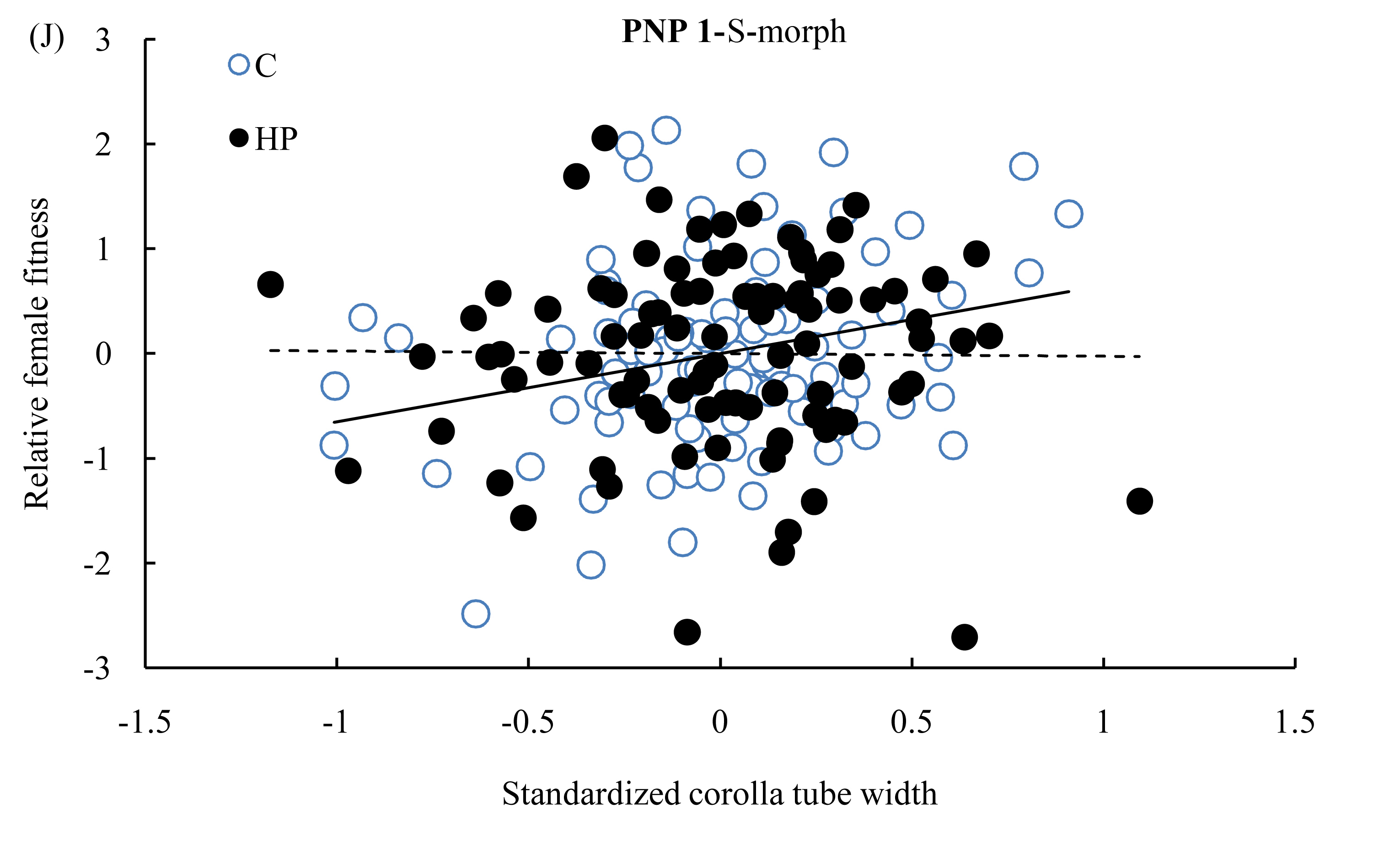


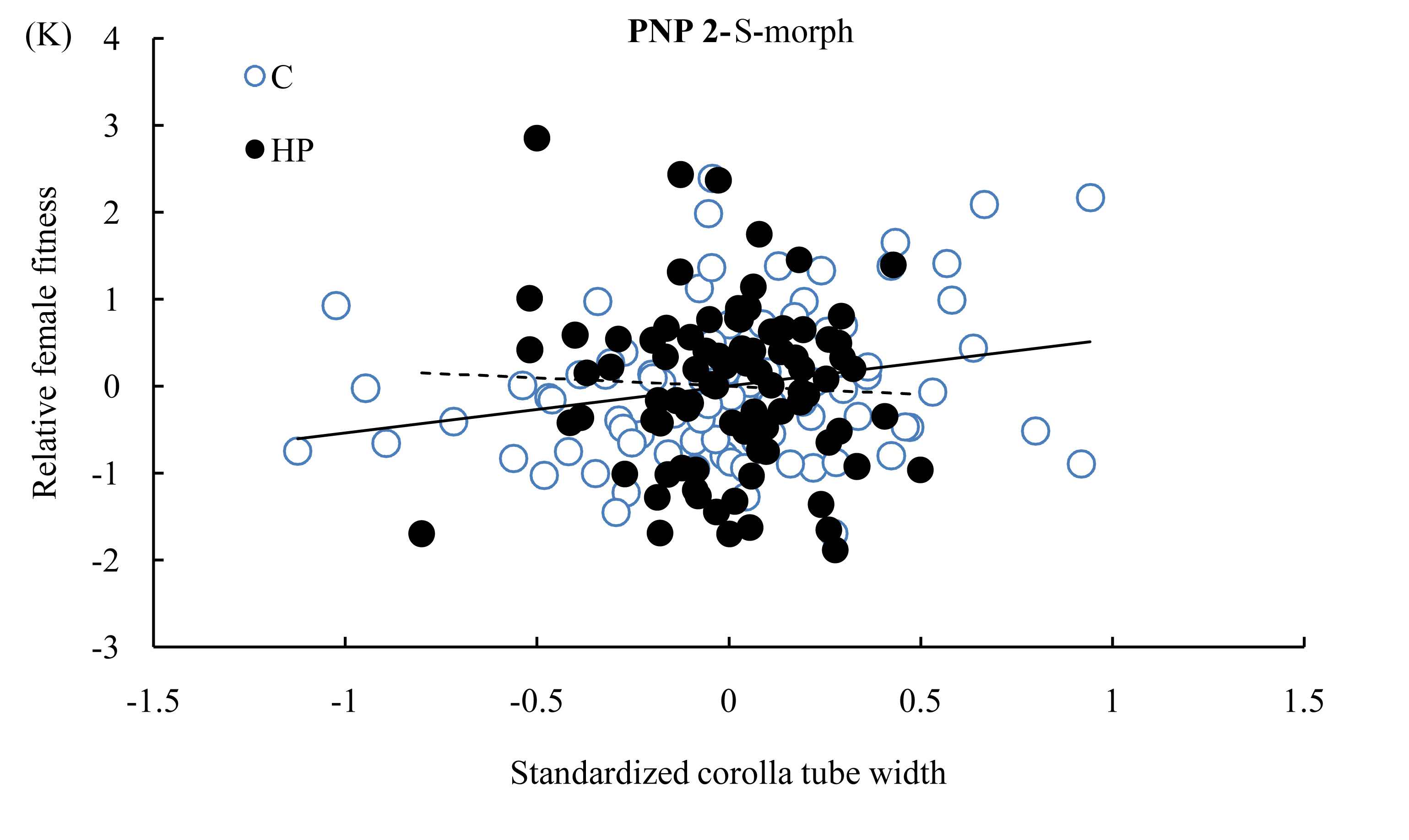


**Figure S3.** Standardized linear phenotypic selection gradients for flowering onset, number of flowers, corolla tube length and corolla tube width in open pollination plants (C, open circles, solid line) and in supplemental hand pollination plants (HP, closed circles, dashed line) at BGTC 1 (**A, B, C**), BGTC 2 (**D, E, H**), PNP 1 (**F, I, J**) and PNP 2 populations (**G, K**). The selection gradients were illustrated with added-variable plot, in which the residuals from a linear regression model of relative fitness on all traits except the focal trait are plotted against the residuals from a regression model of the focal trait on the other traits.
